# Endoscopic dacryocystorhinostomy with short-term, pushed-type bicanalicular intubation vs. pulled-type monocanalicular intubation for primary acquired nasolacrimal duct obstruction

**DOI:** 10.3389/fmed.2022.946083

**Published:** 2022-07-28

**Authors:** Yi-Chun Chi, Chun-Chieh Lai

**Affiliations:** ^1^Department of Ophthalmology, Kaohsiung Medical University Hospital, Kaohsiung Medical University, Kaohsiung, Taiwan; ^2^Department of Ophthalmology, National Cheng Kung University Hospital, College of Medicine, National Cheng Kung University, Tainan, Taiwan; ^3^Institute of Clinical Medicine, College of Medicine, National Cheng Kung University, Tainan, Taiwan

**Keywords:** dacryocystorhinostomy, endoscopic dacryocystorhinostomy, pulled monocanalicular intubation, pushed bicanalicular intubation, nasolacrimal duct obstruction

## Abstract

Dacryocystorhinostomy (DCR) has been a primary treatment for adults with nasolacrimal duct obstruction, while the optimal approach and technique remain controversial. With the advancement of endoscopic DCR and the silicone stents, an update of the surgical outcomes and preferable approaches is required. This study aims at comparing the surgical outcomes of endoscopic DCR using pushed bicanalicular intubation (BCI) to pulled monocanalicular intubation (MCI) in adults with primary acquired nasolacrimal duct obstruction (PANDO). Forty five eyes of 45 patients were enrolled, including 22 eyes of 22 patients treated with endoscopic DCR with pulled MCI and 23 eyes of 23 patients with pushed BCI from January 2014 to June 2021. The success rates at stent removal, 1 month and 3 months after removal were 95, 91, and 82%, respectively, in the MCI group, and 100, 87, and 87% in the BCI group. The BCI group had better success rates but failed to reach a significant difference (*p* = 0.49, *p* = 0.67, *p* = 0.24, respectively). After analyzing with binary logistic regression, the implant material was demonstrated as the predictive of surgical success (*p* = 0.045). There was no significant difference in success rates between patients with dacryocystitis and those without dacryocystitis. We conclude that endoscopic DCR with pushed BCI is easily manipulated and has a promising surgical outcome over pulled MCI. Stent indwelling duration as well as history of dacryocystitis have less influence on the success rates.

## Introduction

Since dacryocystorhinostomy (DCR) was modified in the late nineteenth and early twentieth century (1, 2), it has been an effective treatment for adults with nasolacrimal duct obstruction. DCR was categorized into two major approaches, namely external or endoscopic approach, and with various techniques described, such as laser assisted endoscopic DCR and the utilization of different silicone stents. It was previously believed that external DCR had better success rates over endoscopic DCR (3, 4), yet with the improvement of endoscopic technology, endoscopic DCR had been reported a comparable result in recent reports (5, 6). Leong et al. conducted a meta-analysis for DCR outcomes, which showed a success rate ranging from 64 to 100% in external DCR, 84 to 94% in endoscopic DCR, and 47 to 100% in laser-assisted DCR (7).

It has long been debated regarding the best approach or technique. There were randomized studies as well as meta-analysis comparing the success rates of endonasal with external DCR (5, 8), and DCR with or without intubation (9–11). However, few comparative studies focused on the outcomes of endoscopic DCR with different devices and optimal duration of silicone stenting. This study aims to compare the surgical outcomes of endoscopic DCR with pulled monocanalicular intubation (MCI) to pushed bicanalicular intubation (BCI) in adults with primary acquired nasolacrimal duct obstruction (PANDO).

## Materials and methods

We retrospectively reviewed medical records of adult patients diagnosed with PANDO and received endoscopic DCR as treatment with pulled MCI (Monoka^®^, FCI, Paris, France) or pushed BCI (Nunchaku^®^, FCI, Paris, France) from January 2014 to June 2021 at the Ophthalmology Department of National Cheng Kung University Hospital in Tainan, Taiwan. The diagnosis of PANDO was obtained when meeting both complete nasolacrimal duct obstruction *via* lacrimal duct irrigation and the subjective symptoms with epiphora. The pulled MCI is covered by National Health Insurance in Taiwan, while pushed BCI is not. Thus, the tube selection with MCI or BCI was determined by the patient's decision. Patients with minimum of 3 months follow-up after stent removal were included. Exclusion criteria included patients with trauma of lacrimal system, lacrimal system tumor, abnormal lid position, early silicone tube loss, follow-up duration <3 months and inadequate record information. All procedures were performed by the same ophthalmologist, Dr. Chun-Chieh Lai. Informed consent was obtained from all enrolled patients before invasive interventions performed. This study was approved by an institutional review board at National Cheng Kung University Hospital and in accordance with the ethical standards of the Declaration of Helsinki.

### Procedure technique

**Figure 1:**
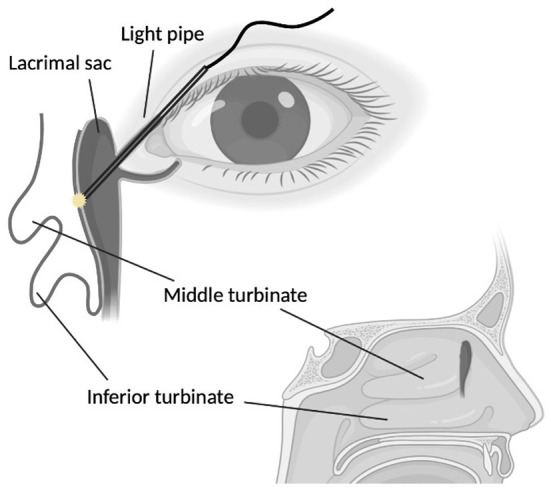
Intraoperative endoscopic dacryocystorhinostomy. The light pipe was inserted from the upper canaliculus into the lacrimal sac. The illumination of lacrimal sac located in front of the middle turbinate.

**Figure 2:**
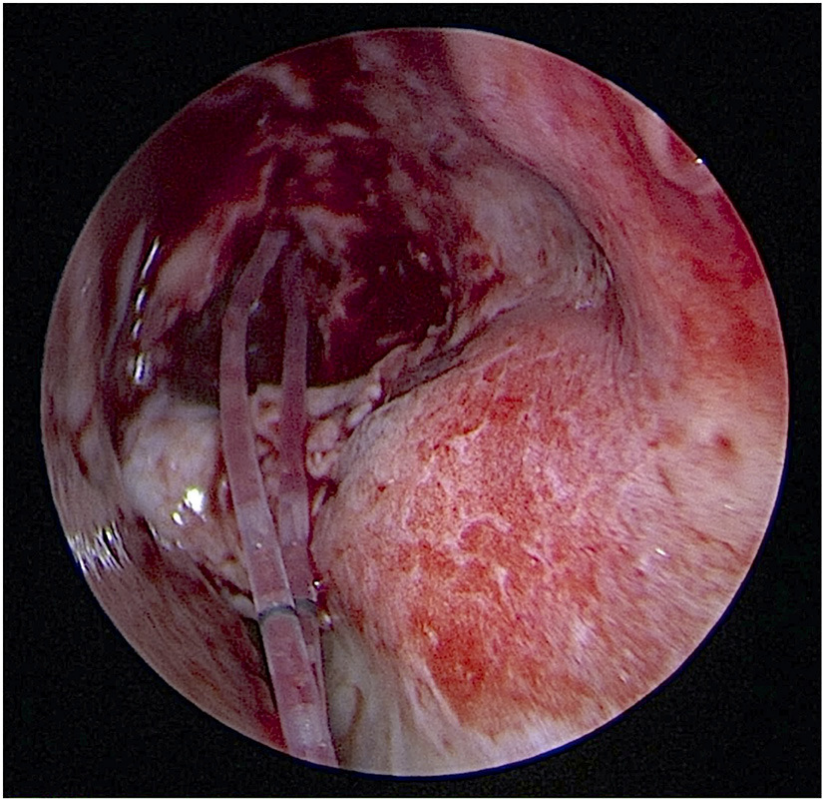
The pushed-type bicanalicular silicone stent (Nunchaku^^®^^) was pushed through the lower and upper pucta into the nasal cavity. No knots or suture were needed for fixation.

All procedures were performed under general anesthesia. We disinfected the nasal cavity and operation site with povidone-iodine, and decongested the nose using 1% lidocaine with 1:100,000 epinephrine. Lacrimal probing was done with a Bowman probe for assessing the patency of lacrimal system. Next, an endonasal endoscopy was performed with 30-degree Karl Storz endoscope. An illuminator, 23-gauge pars plana vitrectomy light pipe, was inserted to the upper canaliculus to help guide the lacrimal sac location, which was usually located along the maxillary line between the frontal process of maxilla and lacrimal bone. The light pipe illumination in the lacrimal sac usually located in front of the middle turbinate from endonasal view (Figure 1). We incised the overlying nasal mucosa from bone under the optic light guide. Thereafter, a bone window was created with a Kerrison bone punch to expose the lacrimal sac. The lacrimal sac was open with relaxing incision. After hemostasis, a pulled MCI or pushed BCI was performed from the canaliculus to nasal cavity. The monocanalicular stent was inserted through the lower canaliculus, pulled into the nasal cavity, and fixed in the punctum. The bicanalicular stent was pushed from lower and upper puncta together with a metallic guide. Noteworthily, no knots or suture were needed to fix the pushed BCI stent (Figure 2). There was no antimetabolite use in our procedure.

### Post-operative care and outcome measurements

All patients were prescribed with topical 0.3% gentamicin and 0.1% betamethasone four times daily for 2 weeks postoperatively, followed by sulfamethoxazole and 0.1% fluorometholone four times daily. The postoperative follow-up was arranged 1 week after the operation, and further appointments were arranged every 2 weeks to every month until the stent removal. The MCI was placed for ~4 months as most previous studies suggested. Due to lack of strong evidence of the intubation duration regarding the new type of pushed BCI, as well as our attempts to relieve the discomfort of stent intubation, BCI was placed with shorter duration around one month. The stents were removed by pulling the monocanalicular stent from the punctum or pulling the bicanalicular stent from the upper and lower puncta under topical anesthesia. After the stents were removed, appointments were arranged monthly to every 3 months, and we performed lacrimal irrigation at every appointment afterwards. The surgical outcomes were evaluated with the irrigation results at the time the stent removed, around 1 and 3 months after silicone stent removal. Patients' reflections after the surgeries were also documented in the medical records. Success was defined as both subjective improvement of epiphora after the surgery and a patent lacrimal system on nasolacrimal irrigation at the outpatient visits. Due to the assumption of independence in the traditional statistics, only one eye was selected in each patient. If both eyes were manipulated and included in current study, the results were defined by the eye with more severe symptoms or with underlying dacryocystitis.

### Statistics

Statistical analysis was performed using software Statistical Product and Service Solutions (SPSS), version 20.00. Data were presented as median (minimum–maximum) in quantitative variables and frequencies in categorical variables. Since the sample size was small and data were not normally distributed, nonparametric tests were applied. Mann-Whitney U test was used for calculating quantitative variables, and Fischer exact test as well as Chi-Square test were applied for comparing the categorical variables. To further examine the relative contribution of potential predictor to the success rate, implant material, stent indwelling duration, age and gender were included in a binary logistic regression model. Adjusted odds ratios (AOR) and the significance of AOR were calculated. *P*-value < 0.05 was defined as statistically significant.

## Results

Sixty one eyes of 53 PANDO patients were reviewed. Five eyes were excluded due to short follow-up duration <3 months, 1 eye due to the traumatic mechanism, 1 eye due to abnormal lid position and trauma-related nasolacrimal duct obstruction, and 4 eyes due to no documented time of stent removal. Five patients had PANDO and treated with DCR with silicone stents in both eyes, and only the eye with more severe symptoms or with underlying dacryocystitis was selected. Forty five eyes of 45 PANDO patients were enrolled in current study. Among them, 22 eyes of 22 patients were performed with endoscopic DCR with pulled MCI, while 23 eyes of 23 patients were with pushed BCI. Characteristics were listed in Table 1. The median age was 63 [23–86] in MCI group and 67 [38–78] in BCI group. There was no significant difference in age (*p* = 0.51), gender (*p* > 0.99) and laterality (*p* = 0.29) between the MCI and BCI group. Stent indwelling duration in the BCI group was significantly shorter than that in the MCI group (*p* < 0.001). Nearly half of the enrolled patients had history of dacryocystitis. Previous lacrimal procedures including balloon dacryocystoplasty (59% in MCI and 78% in BCI group) as well as incision and drainage (27% in MCI and 26% in BCI group) were presented in our study.

**Table 1:** Patient characteristics.

**Variable**	**MCI (*****N** =* **22)**	**BCI (*****N** =* **23)**	***p*** **-value**
	**Mean or N**	**Mean or N**	
Age (min-max) (years)	63 (23–86)	67 (38–78)	0.51^a^
Gender			>0.99^b^
Male	4 (18%)	4 (17%)	
Female	18 (82%)	19 (83%)	
Laterality			0.29^c^
OD	8 (36%)	12 (52%)	
OS	14 (64%)	11 (48%)	
Stent indwelling duration (min–max) (month)	3.90 (2.63–6.70)	1.2 (0.4–1.9)	<0.001^a*^
Medical history		
Dacryocystitis	10	11	
Diabetes mellitus	1	4	
Hypertension	3	6	
Sinusitis	3	0	
NTM infection	0	2	
Head and neck tumor	3	0	
Breast cancer	0	1	
Previous operation			
Balloon	13 (59%)	18 (78%)	
dacryocystoplasty			
Incision and drainage	6 (27%)	6 (26%)	

^a^*Mann-Whitney U test, ^b^Double-tailed Fischer exact test, ^c^Double-tailed Chi-square test*.

The * symbol indicates the statistical significance.

Surgical outcomes were reported in Table 2. Improvement of the symptoms reported by patients and patent lacrimal system with lacrimal irrigation were observed mostly at the visit when the stent removed (95% in the MCI and 100% in the BCI group), and gradually decreased by time. No significant difference was obtained throughout the follow-up. Nevertheless, there was a higher success rate in the BCI group at 3 months after stent removal (87% in the BCI vs. 82% in the MCI group).

**Table 2:** Surgical outcomes of endoscopic DCR with MCI and BCI.

**Variable**	**MCI (*****N** =* **22)**	**BCI (*****N** =* **23)**	**p-value**
	**Mean or Number**	**Mean or Number**	
Success rate
At stent removal	21 (95%)	23 (100%)	0.49^a^
1 month after removal	19 (91%)	21 (87%)	0.67^a^
3 months after removal	17 (82%)	21 (87%)	0.24^a^

^a^*Double-tailed Fischer exact test*.

To evaluate the predictors of surgical success, implant material, stent indwelling duration, age and gender were incorporated into the binary logistic regression model (Table 3). Only the implant material reached a statistical significance (*p* = 0.045) with AOR 39.554 as a predictor of the surgical success at 3 months after stent removal.

**Table 3:** Binary logistic regression analysis of predictors of surgical success in patients with primary acquired nasolacrimal duct obstruction at 3 months after stent removal.

	**Variable**	***B***	**SE**	**Wald Chi-Square**	***p***	**AOR**
Implant	Nunchaku	3.68	1.834	4.022	0.045*	39.554
	Monoka	(Ref)				
Stent indwelling duration		0.692	0.489	1.998	0.157	1.997
Age		−0.041	0.046	0.786	0.375	0.960
Gender	Male	1.106	1.063	1.082	0.298	0.331
	Female	(Ref)				

*SE, standard error; p, p-value; AOR, adjusted odds ratio*.

The * symbol indicates the statistical significance.

We performed subgroup analysis of previous dacryocystitis listed in Tables 4, 5. There was no significant difference between the success rates of endoscopic DCR in PANDO patients with or without previous dacryocystitis either in the MCI or BCI group.

**Table 4:** Surgical outcomes of endoscopic DCR with MCI with or without previous dacryocystitis.

**Variable**	**MCI without dacryocystitis (*****N** =* **12)**	**MCI with dacryocystitis (*****N** =* **10)**	***p*** **-value**
	**Mean or Number**	**Mean or Number**	
Success rate
At stent removal	11 (92%)	10 (100%)	>0.99^a^
1 month after removal	9 (83%)	10 (100%)	0.22^a^
3 months after removal	8 (67%)	9 (90%)	0.32^a^

^a^*Double-tailed Fischer exact test*.

**Table 5:** Surgical outcomes of endoscopic DCR with BCI with or without previous dacryocystitis.

**Variable**	**BCI without dacryocystitis (*****N** =* **12)**	**BCI with dacryocystitis (*****N** =* **11)**	***p*** **-value**
	**Mean or Number**	**Mean or Number**	
Success rate
At stent removal	12 (100%)	11 (100%)	–
1 month after removal	11 (92%)	10 (91%)	>0.99^a^
3 months after removal	11 (92%)	10 (91%)	>0.99^a^

^a^*Double-tailed Fischer exact test*.

There was no immediate complication, such as tube loss, puncta complications or corneal abrasion in our study. Only one patient in the BCI group suffered from post-operative infection of surgical site and failed to maintain a patent lacrimal system at 1 month after stent removal. Some patients had mild nasal bleeding after surgery but controlled easily without the need of additional hemostasis.

## Discussion and conclusion

Endoscopic DCR has gained popularity over the last few decades. It has several advantages over external DCR, namely the cosmetic benefit with no facial scar formation, shortened wound recovery time and hospitalization period, less blood loss, better visualization of endonasal anatomy, the ability to correct endonasal abnormality such as a hypertrophic turbinate and deviated nasal septum simultaneously, and applicability when acute dacryocystitis (6, 10, 12–15). Disadvantages of endoscopic DCR includes more expensive equipment and steep learning curve that a thorough understanding of endonasal anatomy is required (6, 12, 13).

The issue of whether to place stents during endoscopic DCR was frequently discussed. Endoscopic DCR with stents had no significant superiority in success rates over that without stents in previous studies (10, 11, 16). Nevertheless, the recent meta-analysis reported a tendency of improving success rate for endoscopic DCR with silicone intubation after 2012 (17). Due to the rapid recovery, cosmetic benefit and promising outcomes, we performed mostly endoscopic DCR with silicone stents in substitution of external DCR in our institution in recent 7 years.

In current study, PANDO mostly affected our patients around their sixth decades, characterized with female predominant and history of dacryocystitis, which was compatible with previous studies (6, 18, 19). The pathophysiology of PANDO seemed to be multifactorial. It might result from the smaller diameter of nasolacrimal duct in women (20, 21), hormonal change especially in postmenopausal female (22), and derangement of lacrimal drainage-associated lymphoid tissue in chronic dacryocystitis (23).

To manage PANDO, DCR is recognized as the treatment of choice. External approach and endonasal approach with various devices and techniques were reported (12). Rather than using Crawford or Ritleng stent for endoscopic DCR with BCI in previous comparative studies (24, 25), we used a Nunchaku-style silicone tube with a push-to-insert technique. Since the Nunchaku-style silicone tube was introduced by Katsuaki Kurihashi in 1993 (26, 27), it has become another choice of bicanalicular stent other than Crawford stent, which was a more commonly used and historical stent in western countries. The Nunchaku-style silicone tube is characterized by the bilateral thicker tube segments connected with a central rod segment, named after the shape of Nunchaku (26, 27). This design gives the bicanalicular stent a good stability in the lacrimal passage; therefore, there is no need of knots to anchor the stent and consequently it is easy to be removed from puncta. The unnecessity of tying knots avoids the excess tension that might damage or distort the puncta. Noteworthily, there was no BCI dislocation in our study. To date, the Nunchaku-style silicone stent is mostly applied as simple lacrimal system intubation than accompanied with endoscopic DCR in lacrimal duct obstruction. The success rate of solely Nunchaku-style silicone tube intubation was ranged from 63.6 to 95.1% in nasolacrimal duct obstruction in previous studies (19, 27–29).

As illustrated in this study, the success rate of endoscopic DCR with pushed BCI were slightly higher than that in the pulled MCI group at 3 months after stent removal (87 vs. 82%). Moreover, the implant material (the pushed BCI) served as a significant predictor of success. Kashkouli et al. first compared the success rate of endoscopic DCR with pulled MCI to that with pulled BCI using Ritleng stent for adults with nasolacrimal duct stenosis, and reported a similar success rate of 61.53 vs. 59.09%, respectively (25). Andalib et al. reported an equivalent result of 76% success rate in endoscopic DCR with pulled MCI using a Monoka Fayet tube and 76.2% in pulled BCI using a Crawford stent for adults with nasolacrimal duct stenosis (24). The success rates were also reported with no significant difference in the MCI and BCI in congenital nasolacrimal duct obstruction (30–32). The better success rate in pushed BCI might be resulted from the thicker stenting. It was ~0.94 mm of the outer diameter of each thick segment in the Nunchaku stent, in contrast to 0.64 mm of the outer diameter in the Monoka stent. The thicker diameter of the tube provided a lower resistance and increased the lacrimal outflow according to the Poiseuille Law, which states that resistance is inversely proportional to the fourth power of the path radius (33–35). Additionally, the increased outflow may maintain the enlarged passage as named reverbed phenomenon in Moscato's study (35).

In the binary logistic regression, there was no significant difference of the success rate in the stent indwelling duration in current study. The optimal time for stent removal was controversial over time and the recommendation for stent indwelling duration varied from 4 weeks to 6 months. Higher success rate was reported when the stents placed longer in the lacrimal system (36, 37). However, Walland et al. suggested early removal of intubation due to the increased failure rate caused by granulomatous formation when prolonged intubation (38). Charalampidou et al., Kashkouli et al. and Jung et al. stood for an opposite opinion and reported that the timing of tube removal did not influence the success rate (25, 39, 40). Similarly, Zilelioǵlu et al. found no correlation between the indwelling duration and the patency of the lacrimal system (41). Increased risks of complications such as granulation tissue formation of the puncta was reported in prolonged intubation, but good biological tolerance within 6 months of intubation length was documented (39, 41). Our study provided another evidence of early removal of silicone stent at 4–6 weeks might be noninferior and not detrimental. Meanwhile, it might reduce the duration of stent irritation and ease patients' discomfort.

In subgroup analysis, we reported no significant difference of the success rates in patients with or without previous dacryocystitis. Seider et al. revealed a lower success rate in patients with chronic dacryocystitis and treated with external DCR (42). However, recent studies showed a comparable surgical result regardless of dacryocystitis (43, 44). There was no significant difference in surgical outcomes of external DCR in patients with and without history of dacryocystitis reported by Rabina et al. (43). Keren et al. studied the failure factors of endoscopic DCR and reported no correlation between previous dacryocystitis and the success rate (44). Current study is the second one that compared the surgical outcomes of endoscopic DCR with or without previous dacryocystitis other than Keren's study.

To our knowledge, this study is the first comparative literature focusing on endoscopic DCR with pulled MCI using Monoka^®^ and pushed BCI using Nunchaku^®^ with dacryocystitis subgroup analysis in adults with PANDO. Meanwhile, we provide evidence of shorter stent indwelling duration might not be inferior than prolonged stenting, which had been rarely discussed in a comparative study. Our study is also strengthened with one competent surgeon, which minimizes the operator bias.

There are limitations in the current study. First, this study was in a retrospective manner, and the grading of epiphora was not included. There was relatively small sample size that might amplify the statistic bias. Moreover, since most patients with primary acquired nasolacrimal duct obstruction received endoscopic DCR with stenting in our hospital, we compared the outcomes of two stents yet lacked a control group without stent. Furthermore, we studied cases up to 3 months after stent removal. A longer follow-up duration is required to obtain a long-term outcome. Further prospective, large-scaled and long-term study is needed to strengthen the evidence of the potential of endoscopic DCR with pushed BCI.

Conclusively, endoscopic DCR with silicone stenting is an effective surgical approach for patients with PANDO, and the surgical outcomes are not significantly influenced by previous dacryocystitis. The utility of the pushed BCI with Nunchaku^®^ might possess a better surgical outcome than the pulled MCI with Monoka^®^. Early removal of the silicone stent after 1 month is noninferior to prolonged intubation after 4 months, and may reduce biofilm colonization as well as patients' discomfort caused by prolonged stent intubation.

## Data availability statement

The raw data supporting the conclusions of this article will be made available by the authors, without undue reservation.

## Ethics statement

The studies involving human participants were reviewed and approved by Institutional Review Board of National Cheng Kung University Hospital. Written informed consent for participation was not required for this study in accordance with the national legislation and the institutional requirements. Written informed consent was not obtained from the individual(s) for the publication of any potentially identifiable images or data included in this article.

## Author contributions

C-CL conceptualized, designed the study, reviewed, and revised the manuscript. Y-CC collected and analyzed the data, drafted, and revised the manuscript. All authors approved the final manuscript and agreed to be accountable for all aspects of the work.

## Conflict of interest

The authors declare that the research was conducted in the absence of any commercial or financial relationships that could be construed as a potential conflict of interest.

## Publisher's note

All claims expressed in this article are solely those of the authors and do not necessarily represent those of their affiliated organizations, or those of the publisher, the editors and the reviewers. Any product that may be evaluated in this article, or claim that may be made by its manufacturer, is not guaranteed or endorsed by the publisher.
